# Are Circulating Type 2 Vaccine-derived Polioviruses (VDPVs) Genetically Distinguishable from Immunodeficiency-associated VDPVs?

**DOI:** 10.1016/j.csbj.2017.09.004

**Published:** 2017-10-12

**Authors:** Kun Zhao, Jaume Jorba, Jing Shaw, Jane Iber, Qi Chen, Kelley Bullard, Olen M. Kew, Cara C. Burns

**Affiliations:** a Division of Viral Diseases, National Center for Immunization and Respiratory Diseases, Centers for Disease Control and Prevention, Atlanta, GA 30329, USA; b IHRC Inc., Atlanta, GA 30329, USA; c Task Force for Global Health, Decatur, GA 30030, USA

**Keywords:** Vaccine-derived poliovirus, Poliovirus, Polio eradication, Immunodeficiency, Outbreak detection, Comparative genomics

## Abstract

Public health response to vaccine-derived poliovirus (VDPV) that is transmitted from person to person (circulating VDPV [cVDPV]) differs significantly from response to virus that replicates in individuals with primary immunodeficiency (immunodeficiency-associated VDPV [iVDPV]). cVDPV outbreaks require a community immunization response, whereas iVDPV chronic infections require careful patient monitoring and appropriate individual treatment. To support poliovirus outbreak response, particularly for type 2 VDPV, we investigated the genetic distinctions between cVDPV2 and iVDPV2 sequences. We observed that simple genetic measurements of nucleotide and amino acid substitutions are sufficient for distinguishing highly divergent iVDPV2 from cVDPV2 sequences, but are insufficient to make a clear distinction between the two categories among less divergent sequences. We presented quantitative approaches using genetic information as a surveillance tool for early detection of VDPV outbreaks. This work suggests that genetic variations between cVDPV2 and iVDPV2 may reflect differences in viral micro-environments, host-virus interactions, and selective pressures during person-to-person transmission compared with chronic infections in immunodeficient patients.

## 1 Introduction

The Global Polio Eradications Initiative (GPEI), spearheaded by the World Health Organization (WHO), Rotary International, the US Centers for Disease Control and Prevention (CDC), the United Nations Children's Fund (UNICEF) and the Bill & Melinda Gates Foundation, made spectacular success in eradicating polio worldwide [1]. The largest public health program in history [2], [3], [4] has reduced the number of annual diagnosed cases by > 99.9%, from an estimated 350,000 cases in 1988 to 37 wild poliovirus (WPV) cases in 3 countries in 2016 and 12 year-to-date WPV cases in 2 countries in 2017 [1]. Among the three poliovirus serotypes, type 2 poliovirus was declared eradicated in September 2015, with the last virus detected in India in 1999. Type 3 wild poliovirus has not been detected anywhere in the world since November 2012 [1].

GPEI uses two types of polio vaccines, the live attenuated oral poliovirus vaccine (OPV) developed by Sabin and the inactivated poliovirus vaccine (IPV) developed by Salk [1], [2]. Both vaccines are extremely safe and effective. OPV, used for more than five decades to interrupt person-to-person transmission, is the predominant vaccine due to advantages in ease of administration, efficient induction of intestinal immunity, induction of durable humoral immunity, and cost [1], [5], [6]. Due to genetic instability, the live attenuated vaccine-virus in OPV can cause paralysis in extremely rare cases (at a rate of approximately 2 to 4 events per 1 million births [1]) and can also cause the emergence of genetically divergent vaccine-derived polioviruses (VDPVs) [6].

VDPVs are strains derived from OPV, whose ~ 900-nucleotide (NT) sequence encoding the major capsid protein VP1 differs from that of the parental Sabin strain by > 1% for types 1 and 3, and > 0.6% for type 2 [7]. VDPVs are classified into three categories: circulating VDPVs (cVDPVs), when there is evidence of person-to-person transmission; immunodeficiency-associated VDPVs (iVDPVs), shed by individuals with primary immunodeficiencies who have prolonged, sometimes chronic, virus excretion; and ambiguous VDPVs (aVDPVs), which are isolates that cannot be classified as either cVDPV or iVDPV despite thorough investigation. cVDPVs can spread in areas with low vaccination coverage and can cause outbreaks of paralytic poliomyelitis [7]. In contrast to cVDPV, there is no clear evidence for person-to-person transmission and circulation of iVDPV within a community [6].

In April 2016, GPEI implemented a worldwide synchronized switch from trivalent oral poliovirus vaccine (tOPV; types 1, 2, and 3) to bivalent OPV (bOPV; types 1 and 3). The goal of the switch was to prevent the emergence of type 2 vaccine-derived polioviruses (VDPV2s), which have represented > 85% of the VDPV detected since 2000 [8]. After the switch, detection of type 2 poliovirus could indicate continued use of tOPV, circulation of VDPV2, or excretion of iVDPV2 by an immunodeficient person.

It is important to know if a recently identified VDPV is likely to be cVDPV or iVDPV, because public health responses to the two events differ dramatically. Outbreaks require a community immunization response, whereas chronic infections require careful patient monitoring and appropriate individual treatment. If a single sporadic VDPV case could be identified as cVDPV based on the genetic sequence of the excreted virus, this would guide the investigation and the efficient use of limited outbreak resources when planning the scope of a post-switch outbreak response.

This study was designed to investigate whether there is a genetic signal that distinguishes cVDPV from iVDPV. Genetic variation between iVDPV and cVDPV may reflect differences in viral micro-environments, interactions between virus and host, and selective pressures during person-to-person transmission compared with chronic infections in immunodeficient patients [5]. The key properties previously described as useful for differentiating iVDPV from cVDPV are: a) a higher proportion of mixed-base nucleotide sites; b) non-recombinant or vaccine/vaccine-recombinant genomes; and c) the extent of antigenic divergence from the OPV strains [6]. However, systematic quantification of these key properties has not been reported. This study will update recent progress towards the quantification of genetic features distinguishing cVDPV2 from iVDPV2 using publicly available data.

## 2 Methods

We searched for iVDPV2 NT sequences of the VP1 capsid region (903 NTs) from GenBank using the keywords “(immunodeficient or immunodeficiency) and poliovirus”. There were 33 type 2 entries among 402 entries from the initial search as of 6 March 2017. Partial VP1 sequences, reference sequences [9], [10], duplicated sequences and Sabin-like sequences (< 6 NT differences) were removed. Four additional sequences that were not identified in the initial GenBank search were added after detailed literature reviews [11], [12], [13].

For the non-redundant cVDPV2 dataset, we used the keyword “vaccine-derived poliovirus” to obtain 2055 entries from an initial GenBank search, of which 1321 entries were type 2 sequences. Partial VP1 sequences, reference sequences [10], [14], duplicated sequences and Sabin-like sequences were removed.

Data extraction and analysis were performed using MATLAB (MathWorks, Natick, MA). The MATLAB seqprofile tool was used to take multiple aligned sequences as input and to calculate NT frequency profiles for cVDPV2 and iVDPV2 separately. A profile was defined as a matrix (with a size of 4 × 903 in this study) with the frequency of NTs for every column in the multiple alignment. A profile difference matrix was obtained by subtracting the profile matrix for cVDPV2 from the iVDPV2 matrix. The individual profiles and profile difference matrix were visualized in a heatmap.

Sequence logos, graphical representations of the conservation of NTs or amino acids (AAs) at specific sites, were generated using CLC Bio (Aarhus, Denmark). Multiple sequence alignments were calculated using ClustalW [15] and MUSCLE [16]. Transition (Ts) and transversion (Tv) substitutions were counted using Geneious (Biomatters, New Zealand). Total numbers of substitutions for each sequence were obtained by comparison with its parental Sabin 2 strain (accession no. AY184220). Three immunologically important epitopes, termed neutralizing antigenic (NAg) sites (NAg1, NAg2, NAg3), have been described previously [17], [18], [19]. In this study, NAg site locations within VP1 were summarized and assigned to the following amino acid positions: NAg1, 95–103, 168, and 169; NAg2, 219 and 221–223; and NAg3a, 287–289 and 291.

## 3 Results

### 3.1 Dataset Description

A total of 21 unique iVDPV2 sequences from 5 countries (Table 1) and a total of 576 unique cVDPV2 sequences from 10 countries with epidemiological evidence of viral circulation (Table 2), were retained. The cVDPV2 dataset had 6–65 NT substitutions per sequence, whereas the iVDPV2 dataset had 9–154 NT substitutions per sequence, when comparing individual sequences to the parental Sabin 2 strain. A significant proportion in the cVDPV2 dataset had a relative small number of NT substitutions compared to the iVDPV2 dataset. All VP1 sequences used in the analysis were the same length, 903 NTs.

**Table 1:** Non-redundant iVDPV2 dataset.

Country	# of Sequences	Reference(s)
United States	5	DeVries et al., 2011 [11]; Khetsuriani et al., 2003 [30].
United Kingdom	12	Dunn et al., 2015 [21].
Belarus	1	Yakovenko et al., 2009 [12].
Italy	1	Buttinelli et al., 2003 [31].
Libya and Saudi Arabia	2	Schubert et al., 2016 [13].

**Table 2:** Non-redundant cVDPV2 dataset.

Country	# of Sequences	Reference(s)
Afghanistan	7	Sharif et al., 2014 [32].
Cameroon	3	Endegue-Zanga et al., 2015 [33].
Chad	8	Endegue-Zanga et al., 2015 [33].
China	4	Yan et al., 2014 [34].
DRC^a^	76	Gumede et al., 2013 [35].
Egypt	43	Yang et al., 2003 [10]; Blomqvist et al., 2012 [36].
Madagascar	10	Rakoto-Andrianarivelo et al., 2008 [37]; Razafindratsimandresy et al., 2013 [21].
Mexico	4	Esteves-Jaramillo et al., 2014 [38].
Nigeria	409	Burns et al., 2013 [22].
Spain	12	Avellon et al., 2008 [39].

a Democratic Republic of Congo.

### 3.2 Sequence Profiles of VP1 Nucleotides and Amino Acids

**Fig. 1:**
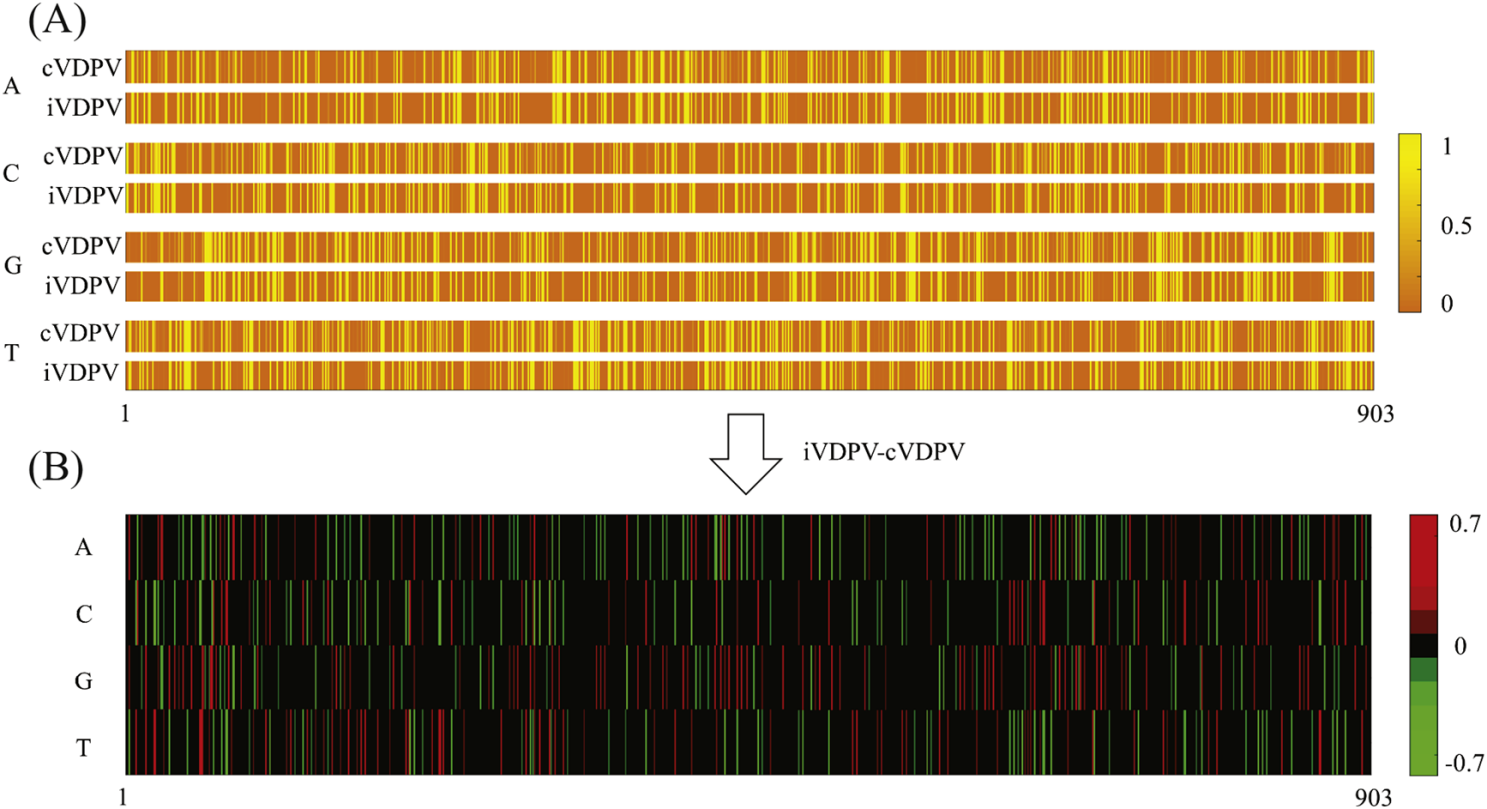
Heatmap for VP1 NT usage. (A) Heatmap color shows the frequency of VP1 nucleotides from position 1 to position 903 (horizontal axis) in a multiple alignment. The vertical axis represents nucleotides and VPVD category (c or i). The scale is from 0 to 1. (B) The frequency difference between cVDPV2 and iVDPV2 (scaled between − 0.7 and 0.7); red vertical lines represent usage of a particular nucleotide that is more utilized in iVDPV2 than cVDPV2 in a particular position, and vice versa for green lines.

To determine whether there are one or more NT positions that can differentiate cVDPV2 and iVDPV2, sequence profiles were calculated from multiple alignments of the cVDPV2 and iVDPV2 sequences. Profiles recording the usage of all four NTs at specific positions (computed as frequencies in percentage) were plotted in a heatmap (Fig. 1A). Similar profiles were observed between cVDPV2 and iVDPV2. Base-by-base and residue-by-residue sequence conservation comparisons using sequence logo plots (Supplemental Figures 1 and 2) showed that no single NT or AA position differentiated one category of VDPV2 from the other.

Subtracting iVDPV2 profiles from cVDPV2 profiles showed that NT preference for the two categories at each position differed within a range from − 0.7 to 0.7 (Fig. 1B). The maximum and minimum preference difference occurred at VP1 position 229, suggesting the strongest NT usage disagreement between iVDPV2 sequences and cVDPV2 sequences at this position. Ninety-nine percent of cVDPV2 sequences and 29% of iVDPV2 sequences had a “G”, whereas 1% of cVDPV2 sequences and 71% of iVDPV2 sequences had an “A”. The preference differences were − 70% (29% minus 99%) for “G” and 70% (71% minus 1%) for “A”, where the positive values implied an iVDPV2 preferred usage and negative values implied a cVDPV2 preferred usage.

A related question is whether there is a NT position that is invariant in Sabin 2 and cVDPV2 sequences but variant in iVDPV2 sequences. Substitution events in a larger dataset, such as cVDPV2, would be expected to be observed more frequently than those from a smaller dataset, such as iVDPV2, if the underlying distributions of substitution events for the two datasets were the same. However, substitutions for iVDPV2 sequences occurred at 16 positions out of 468 invariant positions in Sabin 2 and cVDPV2 sequences (Table 3), confirming greater NT variability in iVDPV2 sequences despite the fact that the cVDPV2 dataset was larger.

**Table 3:** Positions that are invariant in 576 cVDPV2 sequences and Sabin 2 sequence, but variant in 21 iVDPV2 sequences.

VP1 NT^a^ position	NTs in Sabin 2 and cVDPV2 sequences	iVDPV2 sequences	# sequences (iVDPV2) that exhibit variations
32	A	A or C	7
116	A	A or G	1
208	C	A or C	1
218	C	C or T	1
223	T	A or T	12
302	C	A or C	1
307	A	Deletion^b^	1
358	C	C or T	1
433	G	G or T	12
496	A	A or G	1
502	G	A or G	1
506	A	A or C	1
604	G	A or G	1
740	A	A or G	1
761	C	C or T	1
781	A	A or C or T	11C; 1 T

a NT = nucleotide.

b Deletion of a codon (position 306 to 308) was observed in a sequence from [11] (Accession #GU390707).

### 3.3 Pairwise Sequence Identities

**Fig. 2:**
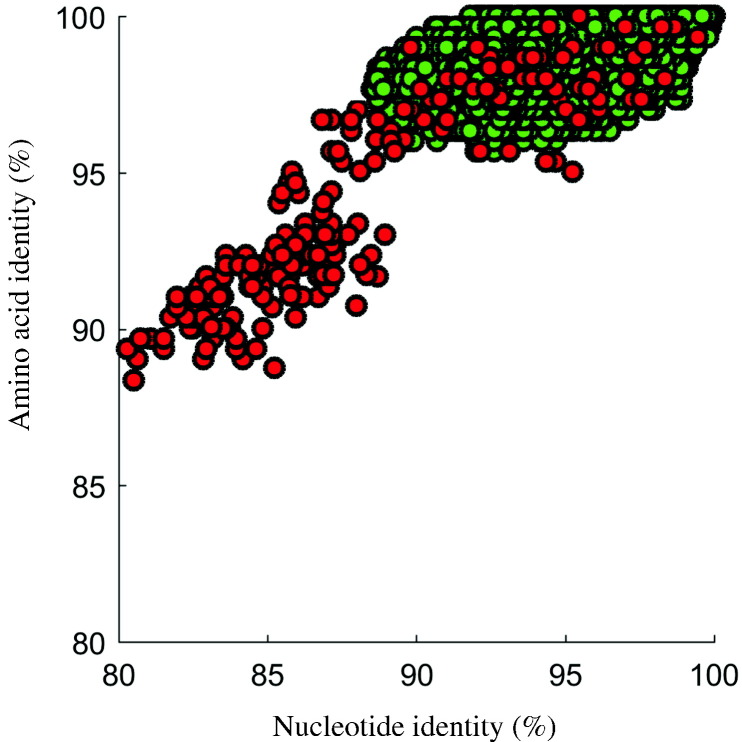
VP1 region analysis of cVDPV2 and iVDPV2 sequence relationships by plotting amino acid sequence identity vs nucleotide sequence identity for all pairs of sequences (576 cVDPV2 and 21 for iVDPV2). Red circles represent iVDPV2 pair-wise comparisons; green circles represent cVDPV2 pair-wise comparisons.

NT and AA sequence identities for the VP1 region were calculated and plotted for all pairs of cVDPV2 (576 sequences) and for all pairs of iVDPV2 (21 sequences), corresponding to 165,600 and 210 pair-wise comparisons, respectively. For a given pair, AA identity was plotted as a function of NT identity [20]. The identity differences in NT and AA between individual viruses in cVDPV2 sequences were bounded, whereas the comparisons among iVDPV2 sequences were dispersed (Fig. 2). Individual sequence pairs for the cVDPV2 dataset differed by up to 11.5% in NT and 4.3% in AA, while the maximum difference of individual sequence pairs for the iVDPV2 dataset was 20% in NT and 11.6% in AA. The upper bound for the difference in cVDPV2 sequences was 99.9% in NT (1 NT substitution) and 100% in AA, whereas the upper bound for the difference in iVDPV2 sequences was 99.4% in NT and 100% in AA.

The pattern in the plot also showed an overlap among values in the two datasets. Slightly less than half of the comparisons in iVDPV2 fell inside the identity area bounded by cVDPV2 sequences (“overlap region”). For example, 142 out of 210 comparisons in iVDPV2 sequences had NT differences greater than 11.5%, whereas 146 out of 210 comparisons in iVDPV2 sequences had AA differences greater than 4.3%. Differentiation between iVDPV2 and cVDPV2 using sequence identity measures was challenging due to the overlap in the two categories at higher NT identity levels (> 88%).

### 3.4 Nonsynonymous Substitutions in the VP1 Region and its NAg Sites

**Fig. 3:**
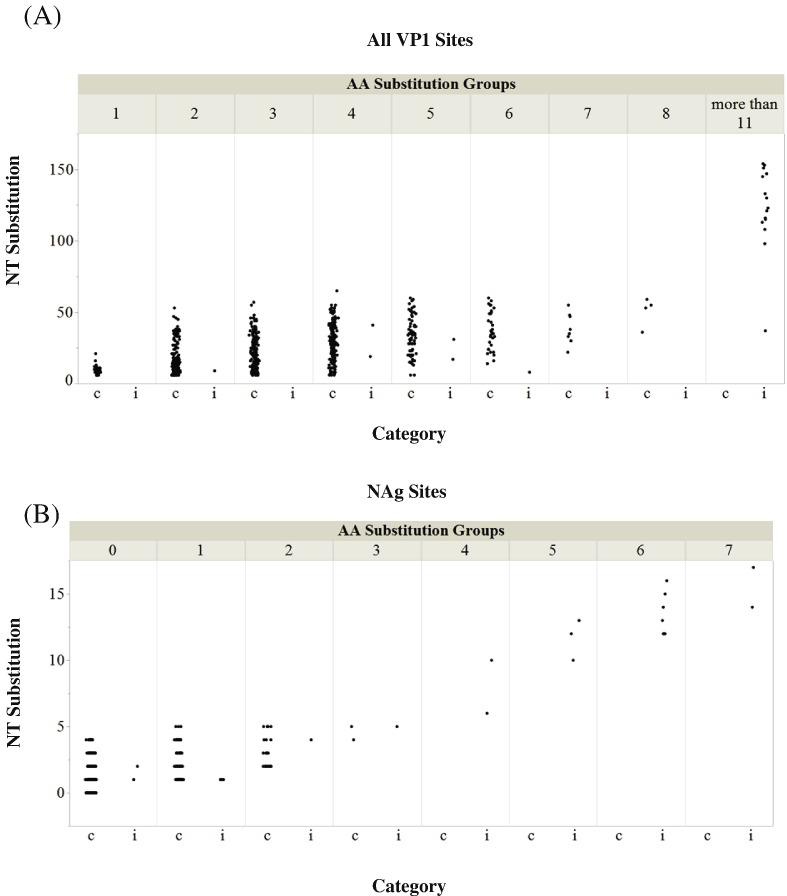
Numbers of NT and AA substitutions in (A) all sites and (B) antigenic sites. Jitter plots were used to prevent overlapping data points in the figure. Distribution of NT substitutions in all sites (A) and antigenic sites (B). The upper horizontal axis represents specific amino acid substitutions groups: VDPV2 sequences in the group labeled “1” have one AA substitution, and VDPV2 sequences in the group labeled “2” have two AA substitutions, etc. Category “c” and “i” on lower horizontal axis represent cVDPV2 and iVDPV2, respectively. The vertical axis represents nucleotide substitution numbers for specific amino acid substitutions groups for either cVDPV2s or iVDPV2s.

A comparison of the distribution of NT substitutions was performed for each category, stratified by the number of AA substitutions (Fig. 3). The numbers of substitutions in the VP1 region and in NAg sites were counted after comparing individual sequences to the parental Sabin 2 strain. Ninety percent of cVDPV2 sequences had fewer than 5 AA substitutions (Fig. 3A). The maximum number of AA substitutions was 8. Seventy-one percent of iVDPV2 sequences had more than 11 AA substitutions, with a maximum of 29 AA substitutions. The numbers may be skewed since 11 of the iVDPV2 sequences were derived from serial samples over a 28-year period from an immunodeficient individual who is a chronic excretor [21]. A similar pattern was observed in the NAg sites (Fig. 3B); there were a maximum of 3 AA substitutions in NAg sites in cVDPV2 sequences and 7 in iVDPV2 sequences. No NT (that encodes AA in a NAg site) or AA substitutions in these sites were observed in 179 cVDPV2 (31%) sequences, but at least one such substitution was found in all iVDPV2 sequences. The distribution of NT substitutions in NAg sites for a given number of AA substitutions showed increased nonsynonymous substitutions in iVDPV2 sequences (Fig. 3B). For example, at a level of 1 AA substitution, the range of NT substitutions for cVDPV2 sequences was from 1 to 5, whereas the NT substitution of iVDPV2 sequences was only 1. The maximum number of NT substitutions in cVDPV2 NAg sites was 5, and was 17 in iVDPV2 NAg sites.

### 3.5 Substitution Patterns, Transition and Transversion

In addition to nonsynonymous substitution analysis, we investigated the Ts and Tv patterns at each VP1 site using Sabin 2 as a reference. If all of the possible pairwise NT substitutions were to occur with equal probability, then the ratio of Ts to Tv would be expected to be 0.5, because there are twice as many possible Tv as Ts. However, Ts was reported about 10-fold more frequently than Tv during poliovirus evolution [22]. As expected, there were more Ts than Tv in this dataset when all substitutions in both cVDPV2s and iVDPV2s were combined (data not shown). The estimated Ts:Tv ratio for the cVDPV2 dataset was 9.4 ± 1.7 whereas it was 4.2 ± 0.6 for the iVDPV dataset (using MEGA 7 [23]).

**Fig. 4:**
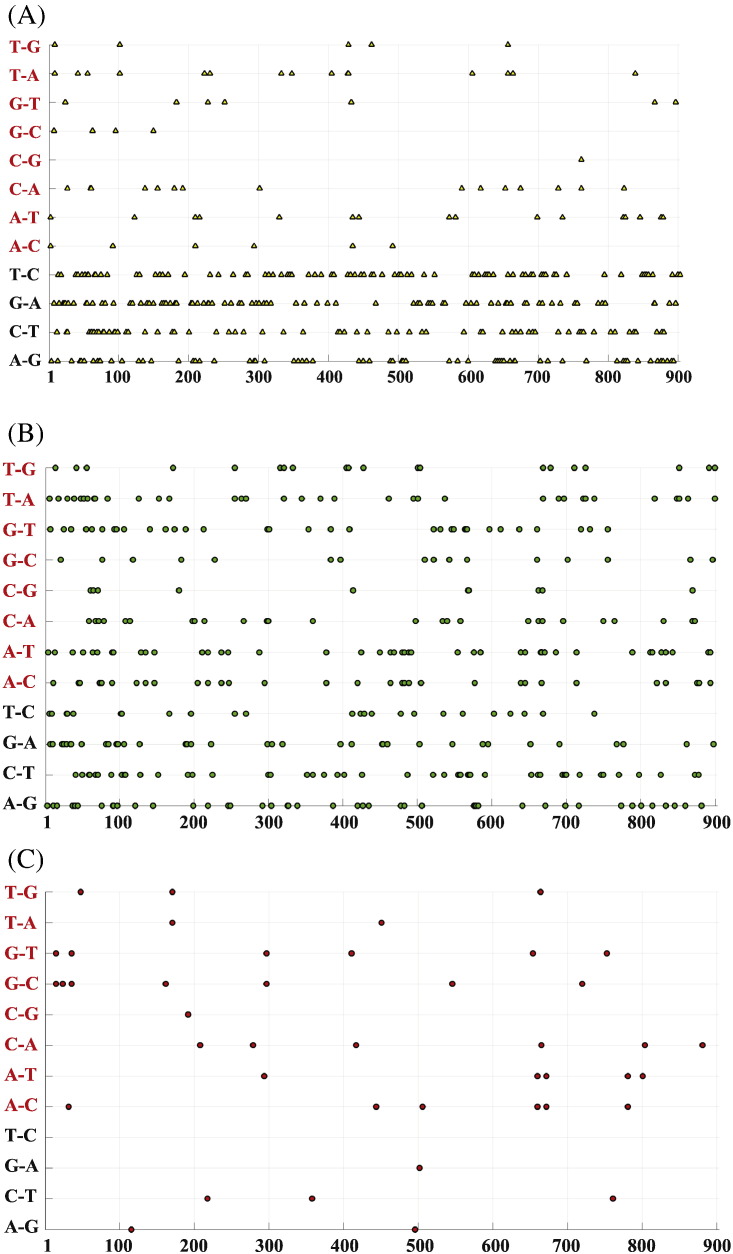
Transitions and transversions. The horizontal axis represents VP1 nucleotide position from position 1 to position 903. The vertical axis represents all possible transitions (in black) and transversions (in red) substitutions. For example, “A–G” means that this position in Sabin 2 is an “A” and is substituted with “G” in at least one VDPV2 in the dataset. (A) Yellow triangles represent the substitution observed in both cVDPV2s and iVDPV2s at the position. (B) Green circles represent substitutions which are uniquely observed in cVDPV2s. (C) Red circles represent substitutions which are uniquely observed in iVDPV2s.

The individual substitutions and substitution types (Tv or Ts) were plotted as a function of VP1 position (Fig. 4). The majority of substitution positions and types observed in the VP1 region were shared across cVDPV2 and iVDPV2. Ts substitution events shared across cVDPV2 and iVDPV2 were more frequent than Tv substitution events at specific positions (Fig. 4A). Interestingly, unique substitution positions and types were more uniformly spread between Ts and Tv in cVDPV2 sequences (Fig. 4B), whereas the substitution types in specific positions in iVDPV2 sequences had more Tv than Ts (Fig. 4C). This may reflect that the frequency of Tv in iVDPV is associated with a higher nonsynonymous substitution rate [22], [24].

## 4 Discussion

### 4.1 Genetic Differentiation Between cVDPV2 and iVDPV2

All isolates that have been determined to be VDPVs are based on VP1 NT sequence variation compared to the parental OPV strain. Classification as iVDPV requires additional clinical testing of a patient's immune status, and classification as cVDPV requires evidence of viral circulation from epidemiology and genetic linkage. In some instances, the NT sequences of isolates are available, but clinical information (for patients' immune status) and/or epidemiological information (for evidence of person-to-person transmission in the community) is not. To date, correct classification of such an isolate using a single NT sequence is not readily available. In fact, there have been no previous studies systematically characterizing cVDPV2 and iVDPV2. To support the investigations required for classification, genetic characterization of the two categories was investigated.

The numbers of NT and AA substitutions in comparative analyses between cVDPV2 and iVDPV2 might be insufficient for a clear distinction between the two categories, although subtle differences between the two categories were amplified by simply subtracting one profile from the other. iVDPV2 sequences have more nonsynonymous substitutions than cVDPV2 sequences. For a given NT identity between any pair of sequences (e.g., 95%) in both cVDPV2 and iVDPV2 categories, iVDPV2 sequences have extended AA divergence compared to cVDPV2 (Fig. 2). These observations suggest that, at best, the signals for distinguishing the two categories are embedded in a combination of positions rather than a single position.

Genetic signals for distinguishing cVDPV2s from iVDPV2s are stronger when comparing more divergent VDPV2 because there is additional time for VDPV2 viruses to accumulate substitutions and to reflect the different microenvironments in the host. In immunocompetent individuals, the presence of virus-specific antibodies resists viral replication. Thus, natural evolution of cVDPV2s is restricted by the host immunologic status. Individuals with primary immunodeficiencies often do not survive without treatment with intravenous immunoglobulin (IVIG), a common treatment in developed countries. Such treatment has been hypothesized to affect the evolution of poliovirus [6]. Since there is lot-to-lot variation in IVIG preparations, and the majority of the iVDPV2 sequences in the dataset were collected in developed countries with greater access to IVIG treatment, it is arguable that individuals were exposed to different antibodies at discrete times, which could drive virus evolution [6], [24], [25].

### 4.2 Implications for the Polio Eradication Endgame

Spearheaded by the GPEI, poliomyelitis eradication is a top global health priority. A key objective of the Polio Eradication Endgame and Strategic Plan 2013–2018 (www.polioradication.org) is the detection and interruption of all poliovirus circulation. Prior to the recognition of VDPV outbreaks, the risk from OPV was considered to be very low, as OPV caused only rare and isolated cases of vaccine-associated paralytic poliomyelitis. However, this notion has changed since the recognition that cVDPV can emerge and cause outbreaks. To prevent cVDPV outbreaks, the WHO planned and implemented the switch from tOPV to bOPV in April 2016 in coordination with all 155 OPV-using countries and territories, with plans to discontinue the use of OPV completely after the eradication of wild poliovirus [26]. Since the switch, detection of VDPV2 has triggered investigations to determine if outbreak response is needed. Because the use of mOPV2 is associated with the risk of triggering new emergences of VDPV2, the Director General of WHO must approve any use of type 2 monovalent oral poliovirus vaccine (mOPV2). WHO has authorized the use of mOPV2 in response to detection of cVDPV2 in Nigeria [27], Pakistan [28] and Mozambique since the global switch to bOPV.

The ability to classify a single VDPV2 sequence as probable cVDPV would allow more effective and timely outbreak response, more efficient use of resources and quicker interruption of outbreaks at their early stages, whereas two or more genetically linked VDPVs are generally required for evidence for circulation. This report not only updates our current understanding of how viral genetic data can inform decision-making in the eradication program, but also highlights the power and limitations of the data. As the molecular epidemiologic data continue to play an essential role and lay the foundation for modeling to inform the public health program, we hope that our findings can help in accurately predicting which VDPVs reflect a poliovirus outbreak.

### 4.3 Limitations of the Dataset and Future Work

This dataset has a ratio of cVDPV to iVDPV of approximately 27:1. This does not precisely reflect the ratios observed through current surveillance, but reflects the fact that iVDPV is much less frequently identified than cVDPV. Reported VDPV cases are rare globally, and the detection frequency of immunodeficient individuals who are excreting VDPV is even lower [6], [8].

Expanded iVDPV2 sequence datasets will be helpful in confirming observations in this study. Additional iVDPV2 sequences are likely to be available in the near future because of ongoing studies designed to identify immunodeficient individuals excreting poliovirus [6]. The immediate next step includes analyzing complete capsid sequences (~ 2600 NTs) from CDC's surveillance dataset to determine whether the larger sequence window (including NAg sites mapped in the VP2 and VP3 regions) will improve resolution. Another step could be to systematically quantify and evaluate the potential use of the presence of recombinant genomes with other species-C human enteroviruses in VDPV isolates, as a possible marker for identifying a VDPV isolate as cVDPV. This step will assess the current understanding that vaccine/non-vaccine recombination frequently occurs in cVDPVs, but is rare among iVDPV isolates [6]. However, this step would require complete genomes; at the present time, WHO only requires labs to collect VP1 sequences.

## 5 Conclusions

The prospect of differentiating cVDPV from iVDPV based on sequence data has attracted increasing attention during the Polio Eradication Endgame [7], [29]. In this study, we presented quantitative approaches using genetic information as a surveillance tool for early detection of VDPV outbreaks. Using publicly available data, we showed that the differentiating task was challenging due to the overlap in genetic characteristics of viruses in the two categories. We also found more pronounced genetic features distinguishing iVDPV from cVDPV when comparing highly divergent VDPVs.

## Competing Interests

The authors declare that they have no competing interests.

## Disclaimer

The findings and conclusions in this report are those of the author(s) and do not necessarily represent the views of the Centers for Disease Control and Prevention.

## Appendix A Supplementary data

Supplementary figures Sequence logo comparisons between cVDPV2 and iVDPV2 using nucleotide and amino acid sequences. Image 1
