# Supplementary figures and images for: Are Circulating Type 2 Vaccine-derived Polioviruses (VDPVs) Genetically Distinguishable from Immunodeficiency-associated VDPVs?
Source: Comput Struct Biotechnol J. 2017 Oct 12;15:456–62. doi: 10.1016/j.csbj.2017.09.004 (PMC5671402; doi:10.1016/j.csbj.2017.09.004)

Supplemental Figure 1.
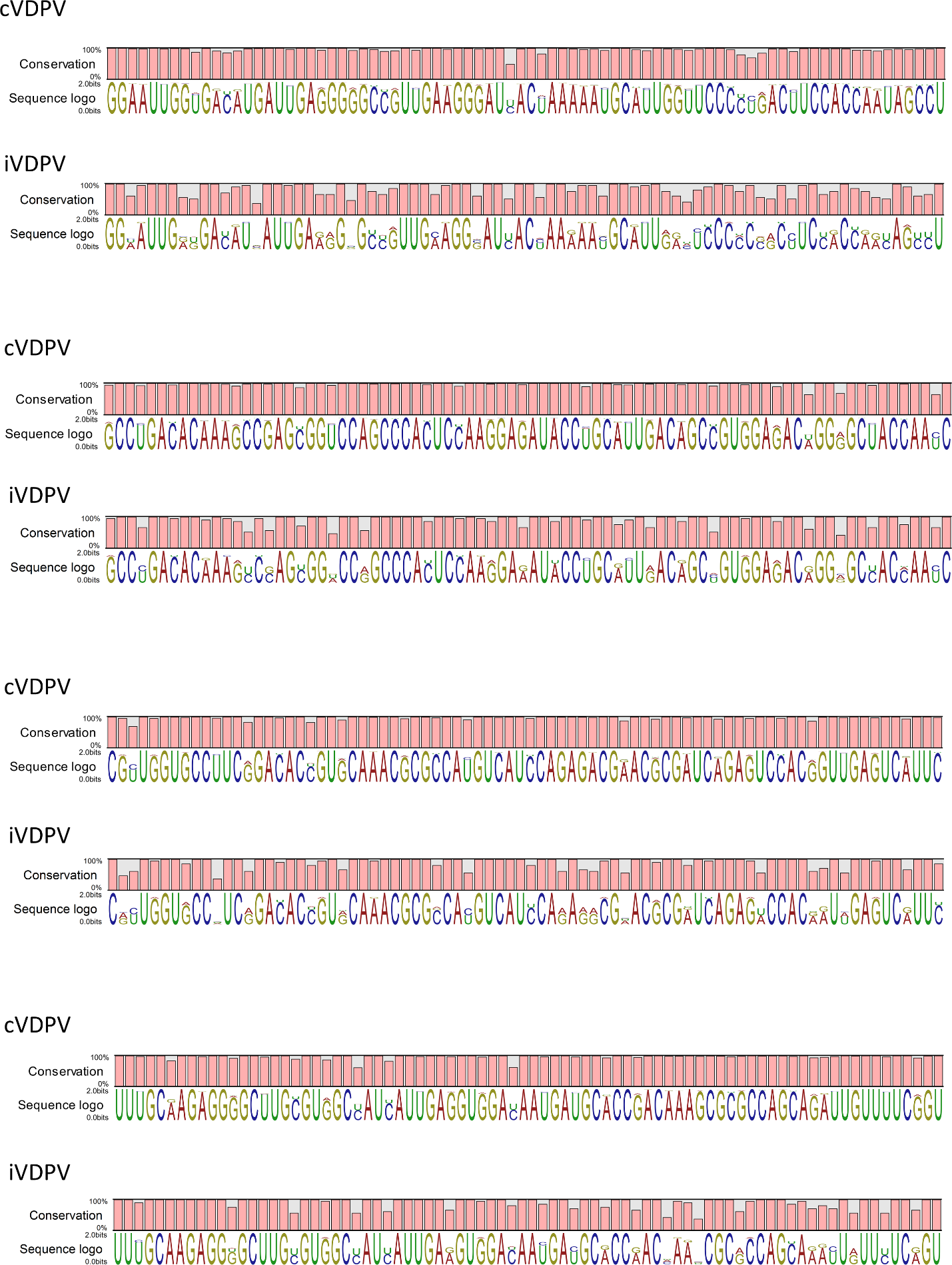

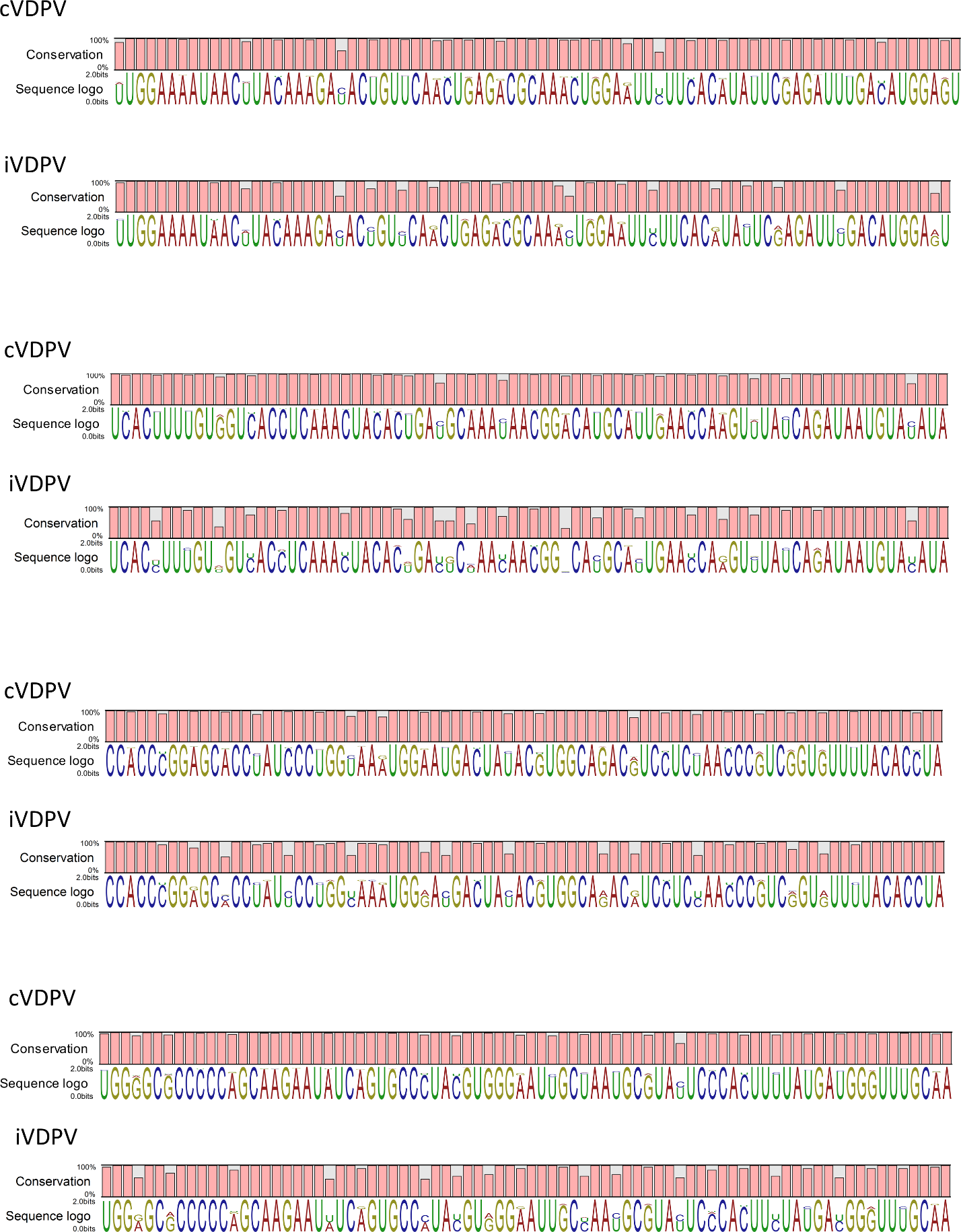

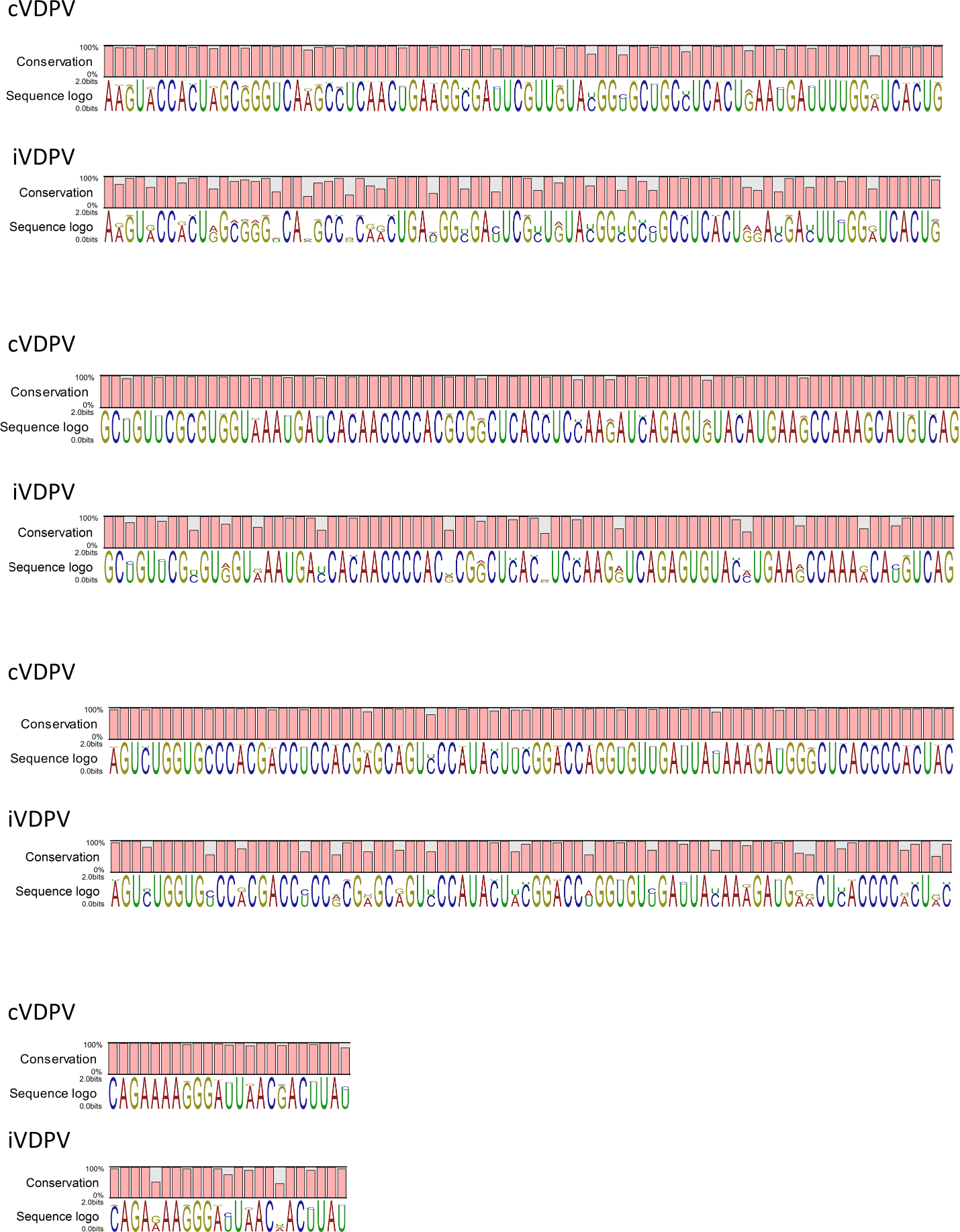


Supplemental Figure 2.


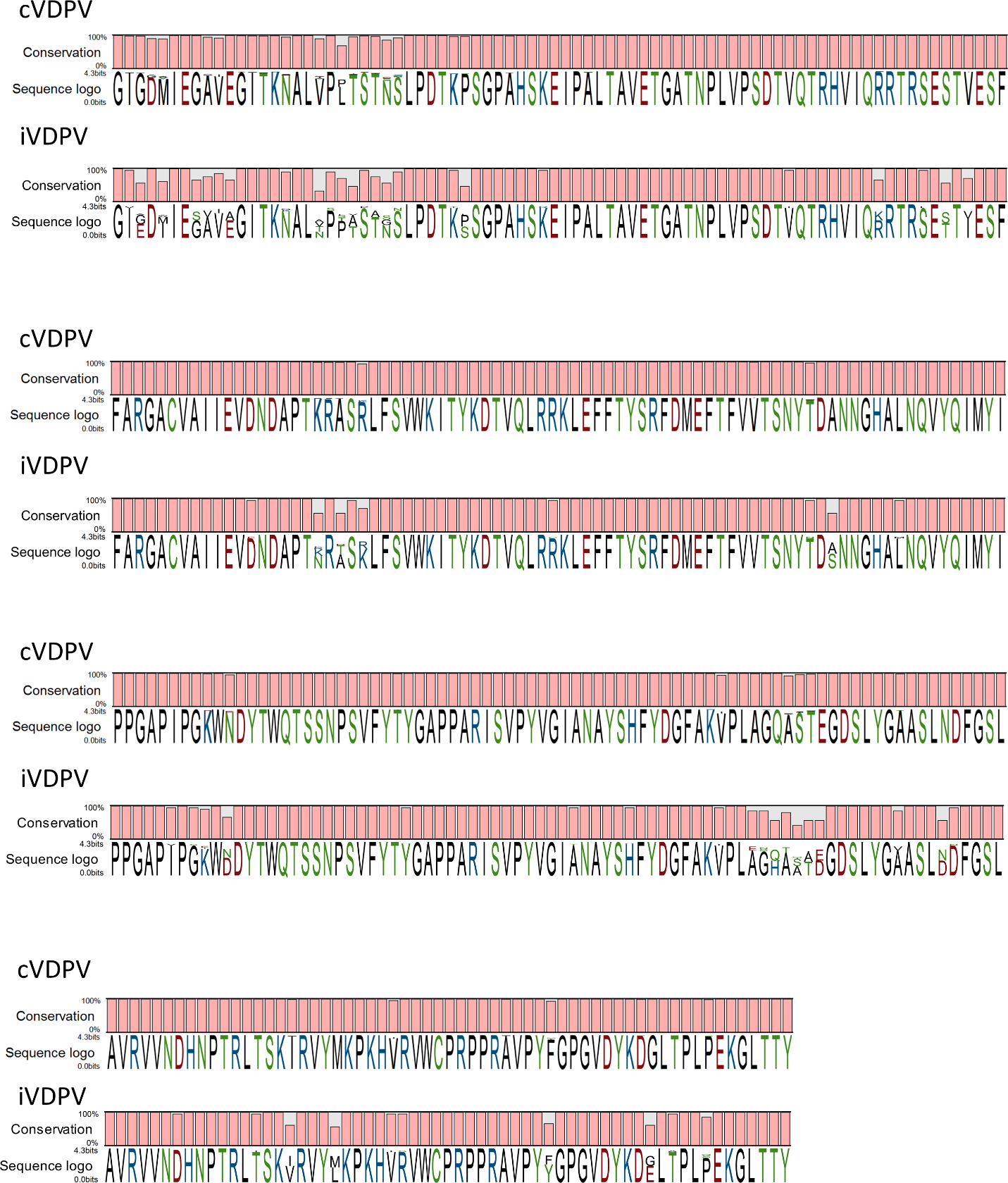

Supplement: Supplementary figures — Sequence logo comparisons between cVDPV2 and iVDPV2 using nucleotide and amino acid sequences. [file mmc1.docx]
